# Prevalence and risk factors of prehypertension and hypertension in Algeria

**DOI:** 10.1186/s12889-022-13942-y

**Published:** 2022-08-18

**Authors:** Abdellatif Moussouni, Adel Sidi-yakhlef, Houari Hamdaoui, Amaria Aouar, Djamel Belkhatir

**Affiliations:** 1 National Center for Prehistoric, Anthropological and Historical Research (CNRPAH, Tlemcen’s station), Algeria, 03, Rue Franklin Roosevelt, 16500 Alger, Algeria; 2 University of Abou Bekr Belkaïd, Algeria. Laboratory of Anthropology, 22, Rue Abi Ayed Abdelkrim Fg Pasteur B. P 119, 13000 Tlemcen, Algeria; 3 University of Abou Bekr Belkaïd, Algeria. Laboratory of Human Actions’ Valorisation for Protection of Environment and Application in Public Health, 22, Rue Abi Ayed Abdelkrim Fg Pasteur B. P 119, 13000 Tlemcen, Algeria

**Keywords:** Hypertension, Prehypertension, Risk factors, Non-communicable diseases, - World Health Organization (WHO) - STEPS, Algeria

## Abstract

**Background:**

Hypertension, also referred to as the silent killer, is known to be one of the most common chronic diseases in the world today. This study aimed to identify the prevalence and risk factors of prehypertension and hypertension among Algerian population.

**Methods:**

This is a descriptive cross-sectional epidemiological study involving individuals aged 18 to 69 years old who were identified in the database of the national survey on non-communicable diseases (NCDs) risk factors conducted in Algeria between 2016 and 2017 using the World Health Organization’s (WHO) STEPwise approach.

Differences in prevalence between normotensives, prehypertensives and hypertensives were assessed using the chi-square test. We also looked at the role of numerous socio-demographic, economic, geographical, and behavioural factors in blood pressure status using a logistic regression model.

**Results:**

The prevalence of prehypertension and hypertension was 36.2% (95% confidence interval: 35.2–37.5%) and 31.6% (95% CI: 30.5–32.7%) respectively. Prehypertension was shown to be substantially higher in males than in women, while hypertension was found to be higher in females compared to men. In addition, both sexes had a rise in the prevalence of blood pressure as they grew older.

A according to multivariate logistic regression analysis, the main common risk factors for prehypertension and hypertension were ageing, obesity, and abdominal obesity. Moreover hypercholesterolemia, and marital status (separated/divorced) were correlated to hypertension.

**Conclusion:**

Prehypertension and hypertension are high and epidemic in Algeria. Therefore, the urgent quantification and monitoring of their risk factors becomes a necessity to plan appropriate preventive measures, in order to fight against NCDs in general.

## Background

High blood pressure (HBP) is one of the five major chronic non-communicable diseases (NCDs) on which the World Health Organization (WHO) has recently published a report calling for effective action. It is a vascular disorder that was initially considered rare- but is now a serious global health problem due to its high morbidity, mortality and management costs. HBP is the most common cardiovascular disease affecting 20% of the world’s adult population and resulting in nine million deaths per year [1]. As a result of population growth and ageing, approximately 972 million people were affected in 2000 [2]. Projections based on these data suggest that by 2025, this number is expected to increase by about 60% to 1.56 billion people worldwide [3].

Many persons with HBP are usually diagnosed accidently or after serious organ damage due to its asymptomatic nature [4]. The level of awareness, treatment and control of the disease remains low in developing countries including Algeria, and as a result, most people affected by HBP are unaware of their status [5].

In a detailed study done on the risks of HBP, the National Committee on Prevention, Detection, Evaluation and Treatment of Hypertension (JNC-7) published its seventh report where they defined prehypertension as the blood pressure (BP) category of 120 ± 139 mmHg systolic blood pressure (SBP), and/or 80 ± 89 mmHg diastolic blood pressure (DBP) [6].

People with prehypertension, according to the same report, have a higher risk of developing hypertension as well as a higher risk of cardiovascular morbidity and mortality [6–8].

The highest worldwide prevalence of hypertension was reported among adults from Africa (with 30% of people affected) followed by Asia and Hispanic origins, compared to Caucasians, while the lowest proportion was recorded in America [9–11].

Algeria, like many other developing countries, is witnessing an increased rate in NCDs, owing primarily to the country’s rapid epidemiological transition over the last three decades [12], as well as the emergence of numerous environmental risk factors as a result of uncontrolled globalization, unrestrained urbanization, and rapid lifestyle change [13].

According to the WHO, NCDs in Algeria accounted for nearly 63% of all deaths in 2010 [14]. In 1993, the epidemiological health survey placed hypertension as the leading cause of consultation (17.2%). The prevalence of hypertension among most Algerian population is around 30–40% [13, 15–17].

Similarly, according to the results of an Algerian STEPwise survey of 4156 people on the measurement of NCD risk factors conducted in 2003,by the Ministry of Health in collaboration with WHO, the proportion of hypertension reached a rate of 26 ± 2.6% [16]. The TAHINA (**E**pidemiological **T**ransition **A**nd **H**ealth **I**mpact in **N**orth **A**frica) study conducted in 2005 among 4818 households also revealed a level of 24.9% [13]. The results of the Africa/Middle East Cardiovascular Epidemiological research for the Algerian subgroup released in 2018with a relatively high estimated rate of 39.5% [18]. In addition, the multi-center Epidemiological Trial of Hypertension carried out in North Africa in 2013 (“ETHNA”), showed a relatively high prevalence of various cardiometabolic risk factors in the Maghreb countries (41.8% in Algeria) with a proportion of hypertension of about 45% (including 29% of new cases) [19].

In sum, the findings of these various national and international studies point to a concerning health situation in terms of the risk of increased morbidity from hypertension and its impact on Algeria’s socio-economic sector.

Although the exact cause of HBP is unknown, several risk factors have been associated with this disease. There are two types: non-modifiable and modifiable variables. Age, gender, race, family history, genetic makeup, and others are non-modifiable risk factors. On the other hand, Obesity, excessive salt intake, physical inactivity, a high-fat diet, smoking, and alcohol consumption, are modifiable risk factors [20].

Overall, genetic and environmental lifestyle determinants play a significant influence in the development of cardiovascular diseases (CVDs) in general and hypertension in particular [13].

At the end, these determinants weigh heavily on patients’ health care costs, especially as chronic diseases require long and expensive management. All these considerations lead us to think about the early diagnosis and the control of BP which has become a requirement for reducing the risk of common health problems, especially CVD.

Thus, it seems that studies on the epidemiology, etiology, and prevalence of prehypertension/hypertension, as well as associated risk factors, are still rare or even less studied in Algeria.

In this paper, we used the descriptive data of the STEPSwise reports in order to assess and explore the prevalence of prehypertension and hypertension in the Algerian population and in biological, demographic and anthropometric risk factor subgroups.

Additionally, we explored these data to identify and analyse the risk factors associated with this disease, aiming to follow the pattern of its course and risk factors and to provide information that can be used as a basis for the design of health policies and programme that would promote a healthy lifestyle and appropriate planning to target preventive public health interventions in Algeria.

## Methods

### Study design and data source

In a detailed study done on the risks of HBP, a secondary analysis was carried out from a descriptive cross-sectional study database of the national survey on the measurement of the burden of NCD risk factors according to the STEPwise Algeria approach, conducted in 2016–2017, by the Ministry of Health, Population and Hospital Reform - General Directorate of Prevention and Health Promotion, in collaboration with the National Institute of Public Health with the support of WHO HQ-Geneva and the WHO- Algeria representation office.

The STEPS is a standardized tool, developed and recommended by WHO for the surveillance of NCD risk factors, morbidity and mortality. They consist of three sequential levels: 1) collecting socio-demographic and behavioral information by pairs of investigators (doctor and health worker) using a standardized questionnaire. This data included: age, sex, locality (rural and urban), education level, marital status and occupation; 2) taking physical measurements of BP, height, weight and waist circumference (WC); 3) performing biochemical tests on blood samples such as fasting blood glucose, blood lipids … [21, 22].

The use of the same standardized questionnaires and protocols allows all countries to use the information produced by this system, not only to monitor trends in their own country but also to make space-time comparisons of indicators (through time and between countries) [21].

The main goal of the STEPwise Algeria survey was to estimate the frequency of these factors on a representative sample of 7450 adult, aged 18 to 69 years old, drawn at random from households throughout the country.

The risk factors studied (STEPS core modules) were tobacco and alcohol use, low fruit and vegetable consumption, physical inactivity, overweight and obesity, HBP, hyperglycemia and dyslipidemia. Optional modules on oral health, mental health, trauma, violence and anti-tobacco policy were also included.

The survey database was obtained from the STEPwise official website [23]. The sampling procedures were described in the survey report [22, 24].

### Definitions and measurements

#### Definition of hypertension

According to the Algerian STEPS survey, HBP is defined as SBP greater than or equal to 140 mmHg and / or DBP greater than or equal to 90 mmHg [25] or patients currently taking antihypertensive medication. An automated BP measuring device (OMRON® digital device) was used to obtain BP readings. Three readings were taken at 3–5-minutes intervals after the participant had rested for 15 minutes. As recommended by the WHO, the average of the last two readings was calculated and used as the final BP measurement [24].

In this study BP was classified according to the JNC7 guidelines [6]. The detection of hypertension was based on self-report of any previous diagnosis of hypertension by a health professional. Participants who were on antihypertensive medication for two weeks were defined as self-reported as well.

Secondly, all individuals who were not diagnosed by health personnel and had an SBP more than or equal to 140 mmHg and/or a DBP greater than or equal to 90 mmHg were considered newly detected hypertensive cases. At the same time, prehypertension was defined as participants not taking antihypertensive drugs and having an SBP of 120 ± 139 mmHg and/or a DBP of 80 ± 89 mmHg. Similarly, normotension was defined as participants with SBP less than 120 mmHg and DBP less than 80 mmHg not taking antihypertensive medication.

#### Definitions of risk factors

The risk factors associated with prehypertension and hypertension were selected and extracted from the STEPS database. They divided into three categories:

1. Socio-demographic variables such as gender, age, education level, professional status, socio-economic level expressed in monthly salary, locality (rural or urban), geographical area,
2. Behavioral variables such as tobacco consumption, eating habits, and physical inactivity. The frequency of alcohol consumption was not included among the risk factors because of the low response rate of respondents in the database, especially women;
3. Clinical variables such as general and abdominal obesity (AO), diabetes, hypercholesterolemia, heart attacks, and heart rate (HR). In addition, for analysis convenience and results interpretation, education level, marital status, geographical region, occupation, smoking status, fruit and vegetable consumption, physical activity, body mass index (BMI) and AO were reclassified and recalculated into original data.

- The education level was classified into five groups: no formal education, primary, middle, secondary and university.
- The marital status was restructured into three categories: married, divorced/separated and widowed.
- The 48 departments surveyed were divided into five geographical regions according to the government division of 1995, Central, East, West, Southern East and Southern West regions [26].
- The wealth index was estimated using the respondent’s disclosed monthly income. The categories were created using the national guaranteed minimum wage as a starting point. This allowed us to divide the people into four groups, ranging from the least wealthy to the richest.
- The weight and height of survey participants were measured using standardized techniques to estimate BMI. It was calculated as weight in kilograms divided by height in square meters (kg/m2).
- The BMI was classified as underweight (<18.5 kg/m2), normal (18.5- < 25 kg/m2), overweight (25 to <30 kg/m2), and obese (30 kg/m2) [27].
- A men WC greater than 94 and a women WC greater than 80 cm was defined as AO [28].
- Physical activity behavior was assessed in three different domains: work, transport and leisure. The activities were divided into two categories: vigorous and moderate. “Vigorous-intensity activities” were those that required a significant physical effort and caused large increases in breathing or HR. “Moderate intensity activities” were those that required only moderate physical effort and caused slight increases in breathing or HR. On this basis, an adult should perform at least 150 minutes of moderate intensity work, 75 minutes of vigorous intensity work, or 60 minutes of mixed vigorous and moderate intensity work per week. Participants were classified as physically inactive if their reported physical activity did not meet the WHO standard [29].
- Fruit and vegetable consumption was divided into two categories following the diet quality indicator: less than five servings per day and five or more servings per day [30].
- Cigarette smoking status was self-reported, with daily smokers, ex-daily smokers, and non-smokers being the three categories.
- The following questions were used to define hyperglycemia, hypercholesterolemia, and heart attacks: “Have you ever been told by a doctor or other health worker that you have high blood sugar or diabetes? “Have you ever been told by a doctor or other health worker that you have high cholesterol?“ and “Have you ever had a heart attack or chest pain from heart disease (angina) or a stroke (cerebrovascular accident or incident)? On this basis, a dummy variable categorized as “yes” or “no” was generated, with the value “yes” depending on the respondent’s answer.

### Statistical analysis

Statistical analysis was performed using Statistical Package for Social Science (SPSS) version 25 (IBM Statistics, USA). Descriptive statistics were used to examine the study characteristics of the population as well as the prevalence of prehypertension and hypertension.

The continuous variables were presented as median (IQR) and compared by the Kruskal-Wallis test after having assessed the normality of the distribution using the Kolmogorov-Smirnov statistical test (p < 0.05).

The categorical variables were expressed as percentages and compared using the Chi-square (χ2) test.

Bivariate logistic regression analysis was performed to identify significant risk factors for prehypertension in association to normotensive status and hypertension in relation with normal BP plus prehypertension. In addition, multivariate logistic regression modelling was performed for all variables of interest showing a significant association with *p*-value <0.05. These variables included age, gender, locality, geographical region, marital status, education level, occupation, behavioral variables such as fruit and vegetable consumption, smoking, physical activity, and metabolic risk parameters such as BMI, AO, hyperglycemia, and hypercholesterolemia which are considered as recommended risk factors for assessment in the STEPS manual [24].

Adjusted odds ratios (AORs) with their 95% confidence intervals (CIs) and *p*-values were generated to determine the predictors of prehypertension and hypertension.

The final multivariate binary logistic regression model was found to be consistent with the results of the Hosmer-Lemeshow fit test.

## Results

### Description of study population

**Fig. 1:**
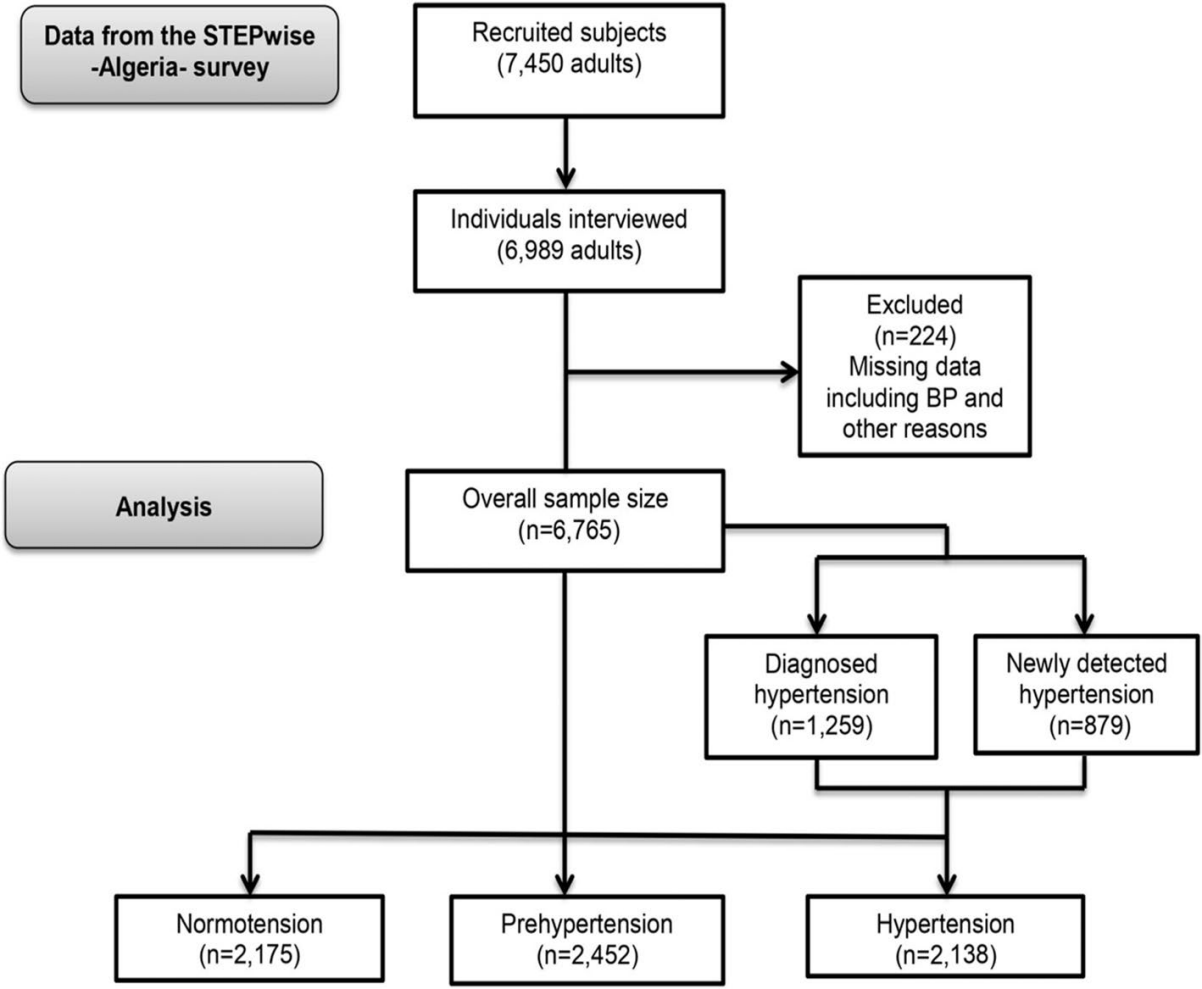
The disposition of overall sample size analyzed

The study was carried out with 7450 adults, of whom only 6989 were interviewed; i.e., a response rate of 93.8% (3082 males and 3907 females, aged 18–69 years). Out of the 6989 participants, 224 were excluded from our study due to missing or outlier data, including BP data (Fig. 1).

Thus, as shown in Table 1, a total of 6765 participants (2968 men and 3797 women) were included in the statistical analysis. With 39.3%, the age group (30–44) is the greatest in the sample, followed by the age group (45–59) with an estimated percentage of 27.7%, the younger group (18–29) with 21.4%, and the older group with 11.6%. Approximately 67.5% of these participants live in urban areas with a predominance of demographic density especially in the northern geographical region of the country compared to those in the south. In terms of marital status of participants in our sample, 69.2% were married, 24.3% single, 3% separated and/or divorced and 3.5% widowed. As for the level of education, 32.5% of participants had a primary school level or have never attended school, while the other three levels (middle, secondary and university) had an almost equal distribution around 22.5%. Homemaker (29.2%) was the most common occupation, followed by the unemployed (24.1%), government employees (17.2%), the self-employed (11.7%), private sector employees (10.4%), and retirees (7.3%). Regarding to the wealth index, 67.9% of respondents earned two times the minimum wage or less (lowest and second lowest), 23.2% earned between two and four times the minimum wage (middle and second highest) and 8.8% earned more than four times the minimum wage (highest).

**Table 1:** Participant characteristics, overall

Predictor variables	Effective (***n*** = 6765)	Percentage (%)
**Gender**		
Male	2968	43.9
Female	3797	56.1
**Age group (years)**		
18–29	1446	21.4
30–44	2659	39.3
45–59	1872	27.7
60–69	788	11.6
**Marital status**		
Never married	1642	24.3
Current married/Cohabitating	4676	69.2
Separated/Divorced	202	3.0
Widowed	239	3.5
**Level of education**		
No formal school	1140	16.9
Primary school	1053	15.6
Moyen school	1600	23.7
Secondary school	1566	23.2
University	1390	20.6
**Locality**		
Urban	4562	67.4
Rural	2203	32.6
**Geographical region**		
North-Centre	2448	36.2
North -West	1470	21.7
North -East	2104	31.1
South -West	215	3.2
South -East	528	7.8
**Occupation**		
Unemployed (able and not able to work) /Student/No paid	1630	24.1
Government employee	1162	17.2
Non-government employee	703	10.4
Self-employed	788	11.7
Homemaker	1974	29.2
Retired	495	7.3
**Wealth index (Family monthly income quintile)**		
Lowest (less than or equal to SMIG)	1172	28.4
Second lowest (More than to SMIG, less than or equal to 2x SMIG	1628	39.5
Midle (More than to 2x SMIG, less than or equal to 3x SMIG	692	16.8
Second highest (More than to 3xSMIG, less than or equal to 4x SMIG)	265	6.4
Highest (More than to4xSMIG)	364	8.8
**Smoking**		
Never smoked	5203	76.9
Past smoked	754	11.9
Current Daily smoked	808	11.1
**Physical activity**		
Low (< 150 Min/week)	1315	19.4
High (More 150 Min/week)	5450	80.6
**Fruits and vegetables consumption**		
< 5 fruit/veg/day	5592	85.1
More 5 fruit/veg/day	978	14.9
**BMI (Kg/m2)**		
< 18,5 (Underwieght)	219	3.3
18,5–24,9 (Normal weight)	2400	36.6
25–29,9 (Overweight)	2303	35.0
≥ 30 (Obese)	1631	24.9
**Abdominal obesity**		
No (Low)	2106	32.2
Yes (High)	4429	67.8
**Blood glucose (Hyperglycemia)**		
Yes	600	17.5
No	2825	82.5
**Cholesterol (Hypercholesterolemia**)		
Yes	665	29.6
No	1578	70.4
**Heart attacks**		
Yes	410	6.1
No	6344	93.9

Furthermore, 24.9% of the Algerian population were obese, 35.01% were overweight, 36.6% were normal, and 3.3% were underweight, according to BMI. AO was found to be present in 67.8% of the population. Tobacco use was distributed between 76.9% of non-smokers versus11.9 and 11.1% of ex-daily and daily smokers respectively. Findings revealed that 85.1% of individuals consumed less than five servings of fruit and vegetables per day. In addition, 80.6% of respondents exercised for 150 minutes or more per week. In addition, 17.5% had diabetes, 29.6% had high cholesterol, and only 6.1% percent experienced a heart attack.

### Prevalence of hypertension, prehypertension and abnormal BP

The socio-demographic, behavioral and health characteristics of participants classified by BP category are presented in Table 2.

**Table 2:** Prevalence of prehypertension and hypertension by predictor variables

Predictor variables	Number of individuals	Normal BP		Prehypertension		Hypertension		Statistic		
		(N)	(%)	(N)	(%)	(N)	(%)	χ2 / Kruskal-Wallis	*df*	***P*** value
		2175	32.2%	2452	36.2%	2138	31.6%			
		CI 95% (31.0–33.3)		CI 95% (35.2–37.5)		CI 95% (30.5–32.7)				
**Gender**										
Male	2968	847	28.5	1282	43.2	839	28.3	110.53 ^a^	2	<0.001
Female	3797	1328	35	1170	30.8	1299	34.2			
**Age group (years)**										
18–29	1446	788	54.5	512	35.4	146	10.1	1281.52 ^a^	6	<0.001
30–44	2659	951	35.8	1120	42.1	588	22.1			
45–59	1872	361	19.3	647	34.6	864	46.2			
60–69	788	75	9.5	173	22	540	68.5			
**Marital status**										
Never married	1642	779	47.4	628	38.2	235	14.3	446.765 ^a^	6	<0.001
Current married/Cohabitating	4676	1307	28	1694	36.2	1675	35.8			
Separated/Divorced	202	57	28.2	69	34.2	76	37.6			
Widowed	239	28	11.7	60	25.1	151	63.2			
**Level of education**										
No formal school	1140	184	16.1	368	32.3	588	51.6	423.42 ^a^	8	<0.001
Primary school	1053	240	22.8	407	38.7	406	38.6			
Moyen school	1600	556	34.8	604	37.8	440	27.5			
Secondary school	1566	598	38.2	585	37.4	383	24.5			
University	1390	589	42.4	485	34.9	316	22.7			
**Locality**										
Urban	4562	1512	33.1	1573	34.5	1477	32.4	18.97 ^a^	2	<0.001
Rural	2203	663	30.1	879	39.9	661	30			
**Geographical region**										
North-Centre	2448	824	33.7	851	34.8	773	31.6	13.24 ^a^	8	NS
North -West	1470	473	32.2	526	35.8	471	32			
North -East	2104	624	29.7	799	38	681	32.4			
South -West	215	74	34.4	76	35.3	65	30.2			
South- East	528	180	34.1	200	37.9	148	28			
**Occupation**										
Unemployed (able and not able to work) /Student/No paid	1630	662	40.6	573	35.2	395	24.2	403.71 ^a^	10	<0.001
Government employee	1162	430	37	438	37.7	294	25.3			
Non-government employee	703	233	33.1	302	43	168	23.9			
Self-employed	788	220	27.9	373	47.3	195	24.7			
Homemaker	1974	564	28.6	615	31.2	795	40.3			
Retired	495	59	11.9	147	29.7	289	58.4			
**Wealth index (Family monthly income quintile)**										
Lowest (less than or equal to SMIG)	1172	344	29.4	415	35.4	413	35.2	8.29 ^a^	8	NS
Second lowest (More than to SMIG, less than or equal to 2x SMIG	1628	524	32.2	586	36	518	31.8			
Midle (More than to 2x SMIG, less than or equal to 3x SMIG	692	230	33.2	249	36	213	30.8			
Second highest (More than to 3xSMIG,less than or equal to 4x SMIG)	265	93	35.1	93	35.1	79	29.8			
Highest (More than to4xSMIG)	364	115	31.6	125	34.3	124	34.1			
**Smoking**										
Never smoked	5203	1741	33.5	1797	34.5	1665	32	98.57 ^a^	4	<0.001
Past smoked	754	161	21.4	291	38.6	302	40.1			
Current Daily smoked	808	273	33.8	364	45	171	21.2			
**Physical activity**										
Low (< 150 Min/week)	1315	406	30.9	457	34.8	452	34.4	5.79 ^a^	2	NS
High (More 150 Min/week)	5450	1769	32.5	1995	36.6	1686	30.9			
**Fruit and vegetables consumption**										
< 5 fruit/veg/day	5592	1790	32	2023	36.2	1779	31.8	0.86 ^a^	2	NS
More 5 fruit/veg/day	978	325	33.2	355	36.3	298	30.5			
**BMI (Kg/m2)**										
< 18,5 (Underwieght)	219	135	61.6	55	25.1	29	13.1	432.65 ^a^	6	<0.001
18,5–24,9 (Normal weight)	2400	966	40.3	930	38.8	504	21			
25–29,9 (Overweight)	2303	627	27.2	888	38.6	788	34.2			
≥ 30 (Obese)	1631	358	21.9	514	31.5	759	46.5			
**Abdominal obesity**										
No (Low)	2106	891	42.3	841	39.9	374	17.8	309.26 ^a^	2	<0.001
Yes (High)	4429	1191	26.9	1536	34.7	1702	38.4			
**Blood glucose (Hyperglycemia)**										
Yes	600	76	12.7	134	22.3	390	65	196.46 ^a^	2	<0.001
No	2825	905	32	938	33.2	982	34.8			
**Cholesterol (Hypercholesterolemia)**										
Yes	665	102	15.3	139	20.9	424	63.8	116.91 ^a^	2	<0.001
No	1578	462	29.3	500	31.7	616	39			
**Heart attacks**										
Yes	410	107	26.1	91	22.2	212	51.7	84.82 ^a^	2	<0.001
No	6344	2063	32.5	2360	37.2	1921	30.3			
**Blood pressure**										
SBP (mmHg) M (P25 ~ P75)		112 (107.5 ~ 116)		127.5 (123 ~ 132.5)		144.5 (134.5 ~ 155.5)		4619.46^b^		<0.001
DBP (mmHg) M (P25 ~ P75)		69 (63.5 ~ 74)		77 (71.5 ~ 82)		82.5 (75 ~ 90)		1912.45 ^b^		<0.001
**Heart Rate**										
HR (bpm) M (P25 ~ P75)		76 (69 ~ 83.5)		77 (70 ~ 84.5)		78.5 (71 ~ 86)		47.23 ^b^		<0.001

Continuous variables were presented as median (IQR) and compared using the Kruskal-Wallis test

Categorical variables were expressed as effectives /percentages and analyzed by the *χ*2 test

Variables are shown as M (P25 ~ P75) or percentage

^a^χ2 test

^b^Kruskal-Wallis test

*df* Degrees of freedom

The prevalence of normotension, prehypertension and hypertension in Algeria was 32.2% (95% CI: 31.0–33.3%), 36.2% (95% CI: 35.2–37.5%), 31.6% (95% CI: 30.5–32.7%), respectively. The prevalence of hypertension was derived from the sum of 18.6% (1259 cases reported to have been previously diagnosed by a doctor or health worker) and 13.0% (879 newly detected cases). The bivariate analysis shows a highly significant difference (*P* < 0.001) between the prevalence of the prehypertension/hypertension groups according to the categories of a) sex mainly due to the high prevalence of prehypertension in men (43.2%) and hypertension in women (34.2%), b) locality which is related to the significantly high proportion of prehypertension in individuals living to rural areas of 39. 9%, c) occupation (due to the high rates of prehypertension among retirees and homemakers as well as the high rate of prehypertension among the self-employed), and d) smoking status owing to the high percentages of prehypertension (45%) and hypertension (40%) among smokers and ex-smokers respectively.

Also, a significantly high association was recorded between hypertension and the last two age groups (45–59); (60–69), the marital status (widowed (63.2%) and separated (37.6%)), the education levels (uneducated, primary with a prevalence of 51.6 and 38.5% respectively), the BMI (overweight and obesity respectively with rates of 34.2% and 46.53), the AO (38.4%), diabetes (65%), hypercholesterolemia (63.8%), heart attacks (51.7%), as well as SBP, DBP and HR (*P* < 0.001).

However, no significant relationship was found between individuals with hypertension compared to participants with normotension and prehypertension in the categories of geographical region, wealth index, physical activity, and fruit and vegetable consumption.

### Risk factors for raised BP (prehypertension and hypertension)

Multivariate logistic regression analysis was performed to determine the factors associated with prehypertension and hypertension (Table 3).

**Table 3:** Risk factors for raised BP (prehypertension and hypertension)

Predictor variables	Normal BP /Prehypertension			Hypertension/ Normal BP + Prehypertension		
	COR (95%CI)	AOR (95%CI)	***P*** value	COR (95%CI)	AOR (95%CI)	***P*** value
**Gender**						
Male	1	1		1	1	
Female	0.582 (0.518, 0.654)	0.382 (0.279, 0.523)	<0.001	1.320 (1.189, 1465)	0.838 (0.529, 1.327)	NS
**Age group (years)**						
18–29	1	1		1	1	
30–44	1.813 (1.574, 2.087)	1.673 (1.265, 2.215)	<0.001	2.528 (2.082, 3.069)	1.965 (1.084, 3.562)	<0.05
45–59	2.758 (2.327, 3.270)	2.286 (1.638, 3.191)	<0.001	7.632 (6.288, 9.263)	4.558 (2.477, 8.388)	<0.001
60–69	3.550 (2.649, 4.758)	2.879 (1.703, 4.868)	<0.001	19.388 (15.439, 24.347)	12.274 (6.041, 24.936)	<0.001
**Marital status**						
Never married	1	1		1	1	
Current married/Cohabitating	1.608 (1.415, 1.826)	0.959 (0.732, 1.255)	NS	3.342 (2.875, 3.885)	1.296 (0.814, 2.063)	NS
Separated/Divorced	1.502 (1.041, 2.166)	1.154 (0.653, 2.041)	NS	3.611 (2.632, 4.955)	2.872 (1.229, 6.712)	<0.05
Widowed	2.658 (1.677, 4.214)	0.928 (0.490, 1.756)	NS	10.274 (7.634, 13.825)	1.732 (0.806, 3.720)	NS
**Level of education**						
No formal school	2.429 (1.961, 3.008)	1.298 (0.882, 1.909)	NS	3.620 (3.051, 4.295)	0.838 (0.503, 1.396)	NS
Primary school	2.059 (1.687, 2.515)	1.079 (0.757, 1.538)	NS	2.133 (1.788, 2.544)	1.009 (0.613, 1.662)	NS
Moyen school	1.319 (1.117, 1.558)	0.890 (0.664, 1.193)	NS	1.289 (1.091, 1.523)	0.810 (0.523, 1.254)	NS
Secondary school	1.188 (1.007, 1.402)	0.860 (0.651, 1.137)	NS	1.100 (0.928, 1.305)	0.762 (0.503, 1.154)	NS
University	1	1		1	1	
**Locality**						
Urban	1	1		1	1	
Rural	1.274 (1.127, 1.441)	1.129 (0.914, 1.396)	NS	0.895 (0.802, 1.000)	1.072 (0.779, 1.477)	NS
**Geographical region**						
North-Centre	1	1		1	1	
North -West	1.077 (0.920, 1.260)	0.899 (0.689, 1.173)	NS	1.022 (0.889, 1.174)	–	NS
North -East	1.240 (1.076, 1.429)	1.144 (0.914, 1.432)	NS	1.037 (0.915, 1.175)	–	NS
South -West	0.994 (0.712, 1.389)	0.813 (0.421, 1.572)	NS	0.939 (0.693, 1.272)	–	NS
South -East	1.076 (0.861, 1.345)	1.254 (0.838, 1.877)	NS	0.844 (0.685, 1.039)	–	NS
**Occupation**						
Unemployed (able and not able to work) /Student/No paid	1	1		1	1	
Government employee	1.177 (0.989, 1.400)	0.539 (0.390, 0.744)	<0.001	1.059 (0.890, 1.260)	0.651 (0.386, 1.099)	NS
Non-government employee	1.497 (1.221, 1.837)	0.653 (0.438, 0.973)	<0.05	0.982 (0.798, 1.208)	0.716 (0.369, 1.390)	NS
Self employed	1.959 (1.603, 2.394)	1.035 (0.686, 1.562)	NS	1.028 (0.844, 1.253)	0.844 (0.435, 1.636)	NS
Home marker	1.260 (1.074, 1.478)	1.073 (0.801, 1.437)	NS	2.108 (1.824, 2.436)	1.286 (0.832, 1.986)	NS
Retired	2.879 (2.086, 3.972)	0.784 (0.464, 1.324)	NS	4386 (3.550, 5420)	0.894 (0.494, 1.619)	NS
**Wealth index (Family monthly income quintile)**						
Lowest (less than or equal to SMIG)	1	–		1	1	
Second lowest (More than to SMIG, less than or equal to 2x SMIG	0.927 (0.770, 1.116)	–	NS	0.858 (0.732, 1.005)	0.826 (0.581, 1.175)	NS
Midle (More than to 2x SMIG, less than or equal to 3x SMIG	0.897 (0.714, 1.129)	–	NS	0.817 (0.668, 0.999)	0.743 (0.489, 1.127)	NS
Second highest (More than to 3xSMIG, less than or equal to 4x SMIG)	0.829 (0.601, 1.143)	–	NS	0.781 (0.585, 1.042)	0.796 (0.461, 1.375)	NS
Highest (More than to4xSMIG)	0.901 (0.674, 1.205)	–	NS	0.950 (0.741, 1.216)	0.981 (0.593, 1.622)	NS
**Smoking**						
Never smoked	1	1		1	1	
Current Daily smoked	1.292 (1.090, 1.531)	0.907 (0.625, 1.316)	NS	0.570 (0.477, 0.682)	0.821 (0.454, 1.486)	NS
Past smoked	1.751 (1.429, 2.146)	1.185 (0.816, 1.719)	NS	1.420 (1.214, 1.661)	1.225 (0.782, 1.918)	NS
**Physical activity**						
Low (< 150 Min/week)	0.998 (0.861, 1.158)	–	NS	1.169 (1.029, 1.328)	1.001 (0.707, 1.416)	NS
High (More 150 Min/week)	1	–		1	1	
**Fruits and vegetables consumption**						
< 5 fruit/veg/day	1.035 (0.879, 1.218)	–	NS	1.065 (0.919, 1.234)	–	NS
More 5 fruit/veg/day	1	–		1	–	
**BMI (Kg/m2)**						
18,5–24,9 (Normal weight)	1	1		1	1	
< 18,5 (Underwieght)	0.423 (0.305, 0.586)	0.306 (0.146, 0.641)	<0.01	0.574 (0.384, 0.859)	0.812 (0.228, 2.889)	NS
25–29,9 (Overweight)	1.471 (1.284, 1.686)	1.210 (0.947, 1.545)	NS	1.957 (1.717, 2.230)	1.039 (0.722, 1.494)	NS
≥ 30 (Obese)	1.491 (1.268, 1.754)	1.362 (1.026, 1.807)	<0.05	3.274 (2.852, 3.760)	1.687 (1.143, 2.489)	<0.01
**Abdominal obesity**						
No (Low)	1	1		1	1	
Yes (High)	1.366 (1.211, 1.542)	1.363 (1.046, 1.776)	<0.05	2.890 (2.545, 3.282)	1.686 (1.119, 2.541)	<0.05
**Blood glucose (Hyperglycemia)**						
Yes	1.701 (1.265, 2.287)	1.351 (0.977, 1.870)	NS	3.485 (2.897, 4.193)	1.341 (0.954, 1.884)	NS
No	1	1		1	1	
**Cholesterol (Hypercholesterolemia)**						
Yes	1.259 (0.947, 1.675)	–	NS	2.748 (2.277, 3.315)	2.029 (1.500, 2.746)	<0.001
No	1	–		1	1	

After adjustment, the analysis revealed that getting older was linked to a linear rise in the probability of having prehypertension. When compared to the younger age group (18–29), this risk was more than one and a half times higher in the (30–44) age group (AOR = 1.673; [1.265–2.215]; *P* < 0.001), more than two times higher in the (45–59) age group (AOR = 2.286; [1.638–3.191]; *P* < 0.001) and about three times higher in the age group (60–69 years) (AOR = 2.879; [1.703–4.868]; *P* < 0.001). Obese individuals who were also abdominally obese had a higher risk of developing prehypertension. When compared to healthy individuals, they respectively have more than one risk in the obese BMI category (AOR = 1.362; [1.026–1.807]; *P* < 0.05) with AO (AOR = 1.363; [1.046–1.776]; *P* < 0.05).

Gender (female) (*P* < 0.001), government employee and non-government employee (*P* < 0.001; *P* < 0.05), and underweight subjects (*P* < 0.01), on the other hand, appeared to have a protective effect.

Prehypertension was not significantly correlated with marital status, education level, locality, geographical region, wealth index, smoking, fruit and vegetable consumption, physical activity, diabetes, and hypercholesterolemia.

Hypertension, on the other hand, was found to have a highly significant relationship with increasing age. When compared to the (18–29 years) group, the risk was almost two times higher in the (30–44 years) age group (AOR = 1.965; [1.084–3.562]; *P* < 0.05), more than four and a half times higher in the (45–59 years) age group (AOR = 4.558; [2.477–8.388]; *P* < 0.001) and about twelve and a half times higher in the (60–69 years) group (AOR = 12.274; [6.041–24.936]; *P* < 0.001). Obese people (AOR = 1.687; [1.143–2.489]; *P* < 0.01) with AO (AOR = 1.686; [1.119–2.541]; *P* < 0.05) had a higher risk of hypertension than healthy people.

Furthermore, marital status, and hypercholesterolemia were all factors contributing to getting hypertension. Separated/Divorced had a risk of hypertension of 2.872; [1.229–6.712]; *P* < 0.05), and patients with hypercholesterolemia had a risk of 2.029; [1.500–2.746]; *P* < 0.001). Gender, education level, locality, geographical region, occupation, wealth index, smoking status, fruit and vegetable consumption, physical activity, and diabetes, on the other hand, were not shown to be significantly associated with hypertension.

## Discussion

Algeria has been the focus of extensive national and international research on evaluating the burden of NCD risk factors in the context of the epidemiological transition study and its impact on health systems in North Africa. In a 2014 WHO report on Algeria, NCDs were associated to 77% of death causes, with CVD accounting for the majority (41%) [31].

Prehypertension and hypertension are the most frequent cardiovascular disorders worldwide, and they are increasingly regarded as one of the most serious public health issues, particularly in developing nations [32].

This study was conducted on a representative sample of 6765 adults using data from the Algerian STEPwise survey 2016–2017 to assess and determine the prevalence of prehypertension and hypertension as well as the risk factors in Algeria.

The results showed that prevalence of prehypertension was high in the Algerian population, with a rate of 36.2%, affecting more males than females (43.2% vs 30.8%). As a result, preventive measures should be adopted with patients who have been diagnosed as prehypertensive, such as monitoring their blood pressure more closely, because a considerable proportion of them are at risk of developing hypertension [6].

Being an under-studied disease in Maghreb countries, prehypertension had a prevalence of 36.2%, which is identical to that found in a cross-sectional survey conducted in Algiers recently which estimated the rate of prehypertension at 36.7% (49.5% in men, against 31.4% in women) [33].

The findings of that study also corresponded to the rate of the adult population in north-east China (36.0%) [34] and the adult population of Brazil (36.1%) [35]. However, it was lower than the rate of Bangladesh’s population aged 25 to 45 years (41.8%) [36], and still slightly higher than the adult population of southern Iran (33.7%) [37] and Taiwanese adults (34%) [38].

Furthermore, the results indicated that the overall prevalence of hypertension in our study was 31.6%, of which 13.0% were newly detected at the time of the study evaluation; i.e., a rate of 41.1% of Algerian participants living with undiagnosed hypertension. The latter may have a higher risk of complications, which would then be too late to avoid.

In comparison to national and regional studies, this proportion of hypertension is consistent with that found in most Algerian populations, which ranges from 30 to 40% [13, 15–17]. It is higher than in some previous studies. The STEPSwise survey in Algeria in 2003 found a BP rate of 26 ± 2.6% [16]. A level of 24.9% was also found in a TAHINA research conducted in 2005 [13]. However, it was still significantly lower than the North African multicenter study conducted in 2013 with a very high hypertension’s proportion of about 45.4% [19], as well as the “Africa / Middle East Cardiovascular Epidemiological” study conducted in 2018 with an estimated hypertension rate of 39.5% for the subgroup “Algeria” [18].

In addition, the prevalence of hypertension in this study was comparable to the overall prevalence in sub-Saharan Africa, which was estimated at 30% [39], as well as Tunisia’s prevalence of 30.6% [40], and higher than the global adult population’s prevalence of 26.4%, Sudan’s prevalence of 27.6% [41], Palestine’s prevalence of 27.6% [42], and Canada’s prevalence of 27% [43].

On the other hand, it was judged to be significantly lower than in Morocco (39.8% [44]), Oman (41.5% [45]), and European countries (38% in Sweden, 42% in England, 47% in Spain, 55% in Germany) [43].

Furthermore, the prevalence of prehypertension in both sexes decreased in the older age groups from 30–44 years, according to our results. However, in both sexes, the prevalence of hypertension rose with age, which is in line with the findings of other research [34, 35].

### Risk factors

Algeria has seen an epidemiological transition as a result of ageing combined with changes in lifestyle, particularly dietary and behavioral patterns. These had a significant impact on risk factors as well as on the incidence of cardiovascular morbidities, diabetes and obesity [12].

Although the bivariate analysis showed a significant correlation between the prevalence of the prehypertension/hypertension groups according to the categories of gender, age, marital status, education level, locality, occupation, BMI (overweight and obesity), AO, smoking status, diabetes, and hypercholesterolemia, the multivariate analysis using logistic regression retained only some determinants with a significant influence on the prevalence of prehypertension and hypertension.

It revealed a highly significant relationship between the chance of acquiring prehypertension and hypertension as one gets older. These results are in line with previous publications from African and global populations [19, 46–48]. The scientists speculate that this is related to changes in blood vessel physiology as people get older. The elasticity of the artery walls would be lost, resulting in a rise in blood pressure [49, 50]. According to previous studies [51], this could be linked to hormonal changes in both sexes at different ages. As a result, frequent monitoring is essential for detecting hypertension early in the menopausal transition.

Furthermore, the results of our study show that obesity is associated with a high prevalence of prehypertension and hypertension. Data from the literature in North Africa [19, 52] and around the world [46, 53] significantly corroborated this conclusion.

This link was frequently discovered among women [35, 46], and as a result, it has become a major public health issue, particularly in developing nations. As a result, WHO classified it as a “global pandemic” Weight gain as a result of improved living standards and decreased physical activity results in increased blood flow to various vital parts of the body like organs and tissues in response to their increased metabolic demands. As a result, the artery walls will be subjected to increased pressure [49]. However, data from Tanzania and Uganda revealed that the population with a high BMI and central obesity had a low proportion of hypertension [54].

Furthermore, our results showed that there was a correlation between the prevalence of AO and the risk of developing prehypertension and hypertension. This matches respectively the findings of Mammeri *et al.* [12] and Midha *et al*. [55]. It appears to be linked to the mechanisms of sympathetic nervous system hyperactivity and renin-angiotensin system activation, according to some authors. By causing peripheral vasoconstriction, these mechanisms can cause hypertension. Dyslipidemia and metabolic dysregulation caused by dietary changes are also risk factors [56, 57]. Consumption of saturated animal fats and processed carbohydrates has increased in recent years, but consumption of fruits and vegetables has dropped [19].

Hypertension in Algeria’s population was found to be positively correlated with marital status (separated/divorced), as in many previous studies. The authors [19, 58] have extensively reported on these findings. It is noted that separated or widowed people compared to married people are more susceptible to have diseases such as anxiety and depression, which explains the risk of developing HBP. Hypercholesterolemia was also a factor in the development of hypertension in our study. This result supports the findings of a number of researche [19, 40, 59]. The direct reasons for this affinity are attributed to urbanization and the nutritional transition associated to the risk of metabolic syndrome [60].

On the other hand, no association was found between the proportion of prehypertension and hypertension with respect to gender, and there was even a protective effect of female versus male gender in prehypertensive status. This result is in line with those mentioned by: Temmar *et al.* [17] and Ong *et al.* [61], who found no difference in hypertension prevalence between the sexes. This could be owing to both sexes having the same economic level. In contrast, Pereira *et al.* [62] discovered a higher average prevalence of BP in males than in women, particularly in developed countries. The gender differences could be explained by the molecular mechanisms underlying vascular, nervous system and kidney functions, that led to hypertension [63], while some studies from sub-Saharan Africa [61, 64, 65] found that the frequency of hypertension is clearly more prevalent for women than for men, with the hormonal profile and postmenopausal status of women accounting for this difference [66].

Furthermore, our study did not show a significant association between geographical region, locality and hypertension. Several studies [67, 68] have noted that people living in urban areas had a higher risk of hypertension. This could be interpreted as a change of lifestyle in these urban areas, including dietary habits, like access to fast food, high-fat, and energy-dense frozen food, and the availability of transport, all of which contributed to physical inactivity. Other studies [19, 34], established the link with the rural area. This is most likely due to the lack of screening as a result of the limited health infrastructure and the low level of education in these areas.

Similarly, we found no evidence of a link between hypertension and education, income, or occupation. These socio-demographic and economic determinants were frequently considered as contributing factors to the development of hypertension [69–71]. According to several studies, illiterate or less educated people, as well as a lack of resources, might had an impact on their general knowledge of how to prevent hypertension which led to unhealthy lifestyles.

In terms to diabetes, the present study found no association with hypertension, contrary to what was pointed out in several research that demonstrated a strong link between the levels of insulin secreted and BP [17, 53].

Although smoking, low fruit and vegetable consumption and physical inactivity play an important role in the development of cardiovascular events including prehypertension and hypertension [72, 73], our results contradicted this while some authors [74] reported the same outcome. The difference could be explained by a number of factors, on top of which is Algeria’s population low proportion of smokers and ex-smokers.

Second, the Mediterranean diet, which is described by several authors as balanced and helpful in improving blood glucose and cholesterol levels in metabolic syndrome, and in preventing diabetes [75].

Third, there has been an upsurge in physical activity among North Africans in recent years [19, 76].

Finally, this study provided important data on some cardiovascular risk factors linked to Algeria’s high rate of prehypertension and hypertension. On the one hand, it would be useful to spread within Algeria’s population awareness on the risks of CVD in order to detect HBP early and effectively treat people with prehypertension to prevent chronic hypertension. The introduction of healthy habits, therefore, should be encouraged [19].

### Study limitations

This study has some limitations. First, the cross-sectional epidemiological study design used in the Algerian STEPwise survey, although used for its speed, efficiency and suitability for chronic diseases, it may not establish the causal relationship between risk factors for prehypertension and hypertension, or may it allow for the generalization of results to the whole population.

Second, the hypertension real prevalence may be inaccurate, either because of under- or over-reporting resulting from self-reporting of hypertension, or because of slight over- or underestimation due to the three measurements of systolic and diastolic BP which were taken only once on one day, or because participants could have recall bias, leading to redundancy in the information provided. Similarly, the study did not include people over 69 years old, which may also distort the overall prevalence of HBP in the population.

Third, the variables analysed in this study can only explain part of the disease determinants.

At the end, the link of genetic factors to the high prevalence of prehypertension and hypertension was not studied.

## Conclusions

The present study found a high prevalence of prehypertension and hypertension in the adult Algerian population by analysing data from the STEPwise Survey (2016–2017). The lack of awareness of prehypertension among many participants is a concerning issue. Those at higher risk of hypertension need special attention to control the future burden of NCDs.

Therefore, prehypertension, hypertension and the majority of their risk factors are frequently preventable or effectively managed through health education about the importance of early detection and preventive interventions. Ways of raising doing this can be in the form of lifestyle awareness targeting diet, physical activity, and smoking cessation, among other things.

Finally, these results are important for establishing a national programme and policies related to the initiation of prevention and public health control in order to reduce the prevalence of NCD in general and to prevent the increase of health care expenditures in the future.

## Data Availability

The survey database was obtained from the STEPwise official website. Those datasets used and/or analysed during the current study are available from the first author Abdellatif Moussouni (abdellatif.moussouni@gmail.com) on reasonable request.

## Abbreviations

AO: Abdominal Obesity
AOR: Adjusted Odds Ratio
BMI: Body Mass Index
BP: Blood Pressure
CI: Confidence Interval
CVDs: Cardiovascular Disease
DBP: Diastolic Blood Pressure
HBP: High Blood Pressure
HR.: Heart Rate
JNC-7: The Seventh report of the National Committee on Prevention, Detection, Evaluation and Treatment of Hypertension
NCDs: Non Communicable Diseases
SBP: Systolic Blood Pressure
SMIG: The national guaranteed minimum wage
SPSS: Statistical Package for Social Science
TAHINA: The Epidemiological Transition And Health Impact in North Africa
WC: Waist Circumference
WHO: World Health Organization
